# Eg5 inhibitor YL001 induces mitotic arrest and inhibits tumor proliferation

**DOI:** 10.18632/oncotarget.17207

**Published:** 2017-04-18

**Authors:** Yufei Wang, Xingyu Wu, Mufeng Du, Xi Chen, Xianling Ning, Hong Chen, Siyuan Wang, Jia Liu, Zhenming Liu, Ridong Li, Ge Fu, Chunguang Wang, Michael A. McNutt, Demin Zhou, Yuxin Yin

**Affiliations:** ^1^ Institute of Systems Biomedicine, State Key Laboratory of Natural and Biomimetic Drugs, Beijing Key Laboratory of Tumor Systems Biology, Department of Pathology, School of Basic Medicine, Peking-Tsinghua Center for Life Sciences, Peking University Health Science Center, Beijing 100191, China; ^2^ State Key Laboratory of Natural and Biomimetic Drugs, School of Pharmaceutical Sciences, Peking University Health Science Center, Beijing 100191, China

**Keywords:** Eg5 inhibitor, antitumor agent, 1,5-disubstituted tetrazole, scaffold hopping, phenotypic screening

## Abstract

Eg5 is a kinesin spindle protein that controls chromosomal segregation in mitosis and is thus a critical drug target for cancer therapy. We report the discovery of a potent, selective inhibitor of Eg5 designated YL001. YL001 was obtained through shape similarity based virtual screening, and it bears a 1,5-disubstituted tetrazole scaffold. YL001 exhibits favorable bioactivity in a variety of cancer cell lines, including taxol-resistant ovarian cancer and 6TG-resistant breast cancer cell lines. This compound inhibits tumor growth by 60% and significantly prolongs median survival time by more than 50% in a xenograft mouse model. YL001 blocks the ATPase activity of Eg5 and causes mitotic failure, ultimately resulting in apoptosis of cancer cells through activation of the caspase-3 pathway. Our findings demonstrate that YL001 is a potent antitumor agent that may be developed for cancer therapeutics.

## INTRODUCTION

The kinesin spindle protein Eg5 (also known as KSP or KIF11) is an important drug target in antimitotic cancer therapy. In early stages of mitosis, Eg5 contributes to centrosome separation through regulation of spindle elongation, which is essential for the formation of the bipolar spindle and segregation of sister chromatids [1, 2]. Successful blockage of Eg5 induces a hallmark phenotype known as the monopolar spindle, and results in cellular mitotic arrest and apoptosis [2, 3]. Overexpression of Eg5 leads to significant instability of the genome in mouse models and carcinogenesis [4]. The generally negligible expression of Eg5 in non-proliferating adult tissues results in a lower toxicity profile of Eg5-targeted therapy as compared to traditional anti-mitotic therapies. As Eg5 is strongly expressed in specific tissues such as blood and lymphatic vascular endothelial cells and is responsible for tumor angiogenesis, inhibition of Eg5 may have both an anti-proliferative effect as well as an anti-angiogenesis effect [5]. Loop L5 of Eg5 is considered to be an important component of the druggable Eg5 allosteric pocket. Among kinesin spindle protein family members this loop is the greatest in length, and thus better accommodates small molecule modulators, therefore providing an ideal structural basis for Eg5 inhibitor specificity [6, 7].

Numerous Eg5 inhibitors have been designed to target this allosteric pocket, and agents are currently undergoing clinical trials [8–15]. ARRY-520, developed by Array BioPharma, is at the forefront of Eg5 inhibitor research and development. The phase III clinical trial of this compound was successfully completed in August, 2015 (data from Thomson Reuters Cortellis^TM^). Another promising Eg5 inhibitor candidate 4SC-205 which is ongoing under Phase I trial can be administrated orally for the treatment of solid tumor and hematologic neoplasia (ClinicalTrials.gov Identifier: NCT01065025). Neoplasm was stabilized in 67% of patients for 100 days with this agent, and median survival time was prolonged. In multiple clinical studies, Eg5 inhibitor treatment has shown favorable characteristics such as high tolerance, good pharmacokinetic/pharmacodynamic properties and low CNS toxicity [14, 16–18]. At the same time, other Eg5 inhibitor scaffolds such as S-trityl-L-cysteine (STLC) and its analogues [19, 20], benzimidazoles [21], thioxoimidazolidines [22], phenothiazanes [23], thiophenes [24], and biphenyls [25] are also in development.

Virtual screening is widely applied for identification of novel targeted drugs. This strategy is generally divided into structure based virtual screening (SBVS) and ligand based virtual screening (LBVS). Although these may appear to be two independent approaches to drug design, in practice they can be utilized together in a mutually advantageous way. Q.D. You pioneered use of LBVS and structure based drug design (SBDD) for discovery of novel Eg5 inhibitors, and this led to the discovery and identification of 1,4-dihydroquinolin-4-ones and 1,2,3,4-tetrahydroquinazolin-4-ones [26, 27]. The first successful application of virtual screening in this field was conducted by F. Kozielski, who combined pharmacophore modeling and molecular docking to identify three novel Eg5 inhibitors bearing quinazoline and thioxoimidazolidine scaffolds [22].

Inspired by these previous adaptations of computer aided drug design (CADD) in the field of Eg5 inhibitor discovery, we further optimized the workflow to search for a new scaffold with antitumor activity. In the first round of virtual screening for potential Eg5 inhibitors, a pharmacophore model (Supplementary Figure 1A, 1B) was combined with *in silico* docking (Supplementary Figure 1C) to identify the compound 7170 which had a moderate degree of antitumor activity (Supplementary Figure 1D). However, this compound does not break away from the triphenylmethyl scaffold of S-Trityl-L-cysteine (Table 1). In view of this scaffold's limited potential for further development, we decided to conduct a second round of virtual screening by scaffold hopping. As the strict shape constraints of the pharmacophore model were likely to limit the ability of virtual screening to break away from the original scaffold, ROCS and EON from OpenEye was selected to perform a 3D similarity search. Multiple studies were used ROCS and EON for successful 3D similarity searches [28], offering an abundant source of material for refining our virtual screening protocol and optimizing the success rate [29, 30].

**Table 1:** EC_50_s (μM) of 3 compounds in enzyme and cell based assays

Compound	Enzyme EC^50^ (μM)	Cell EC^50^ (μM)	KD
7170			
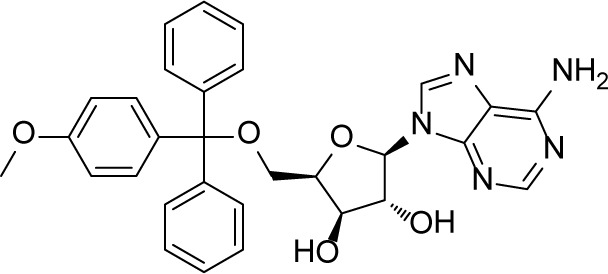	18.03	13.32 ± 1.32	1.131 × 10^−6^
YL001			
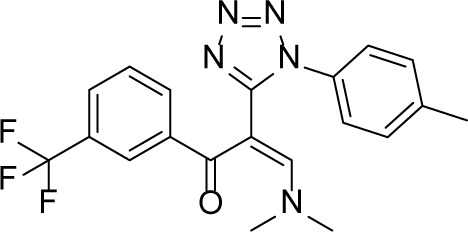	1.18	14.27 ± 0.78	1.327 × 10^−7^
STLC			
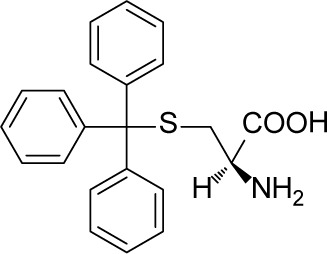	2.52	1.46 ± 0.06	3.767 × 10^−7^

Enzymatic EC_50_s were assessed in Eg5 ATPase activity and cellular EC_50_s were evaluated on HeLa cell line. KD values were calculated with SPR Biacore T200. STLC was used as a control.

The 3D similarity search in the second round of virtual screening resulted in successful scaffold hopping, as the 1,5-disubstituted tetrazole compound YL001 is structurally unique in the field of Eg5 inhibitor research. This agent induces the Eg5-specific monopolar spindle phenotype and displays a strong antiproliferative activity over a broad range of cancer cell lines, including the taxol-resistant A2780 cell line and the 6TG-resistant 4T1 cell line. In a melanoma xenograft mouse model, YL001 inhibited tumor growth by 60% and prolonged median survival time by more than 50%. These findings demonstrated YL001 has the potential for Eg5 targeted therapy.

## RESULTS

### Discovery of YL001 by virtual screening

**Figure 1:**
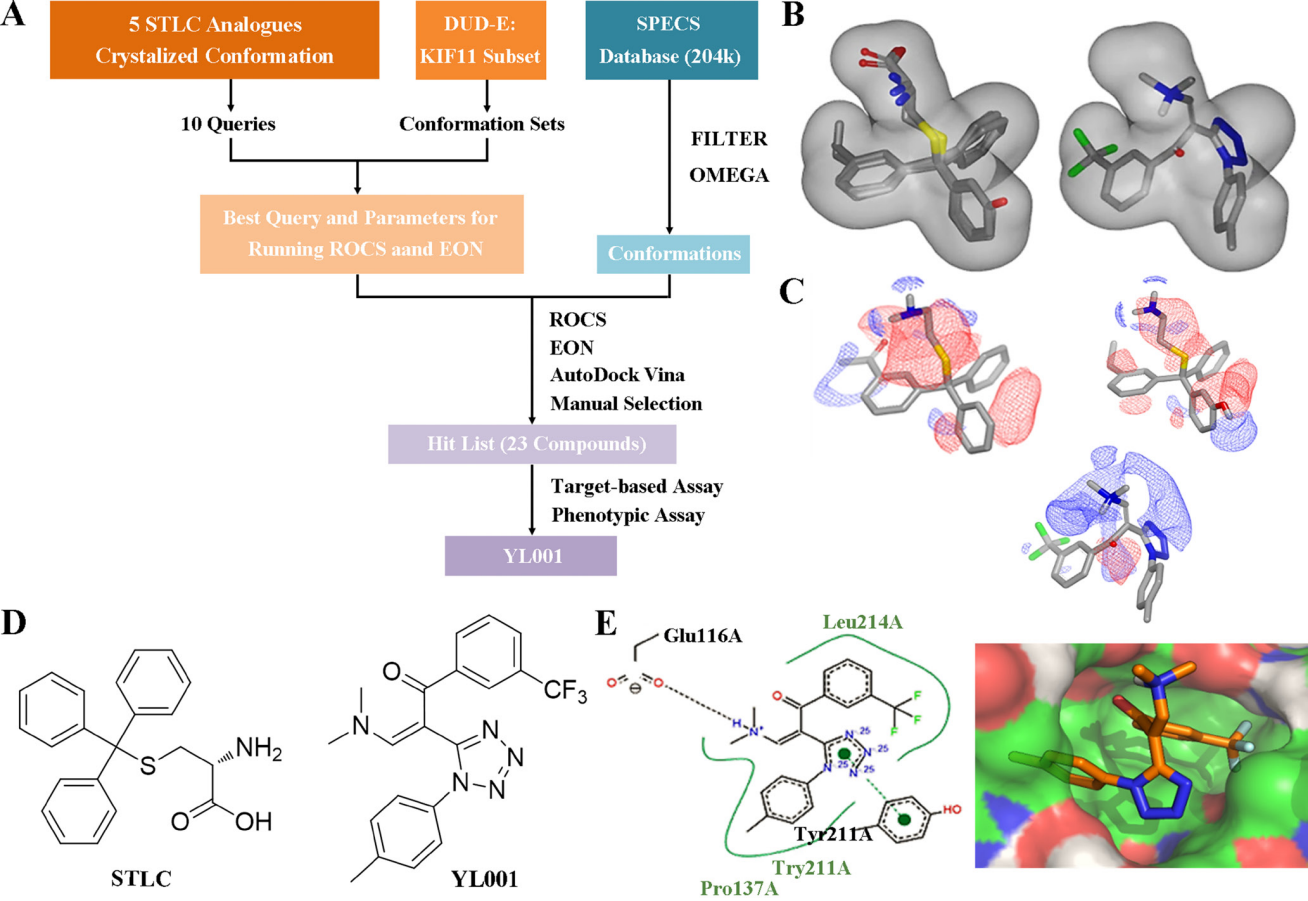
Identification of novel Eg5 inhibitors with 3D similarity search based virtual screening (**A**) Virtual screening workflow. (**B**) Molecular shape comparison of query5 (left) and YL001 (right); grey shape contours in both figures are query5 shape contours. (**C**) Molecular surface electrostatic map showing the ligand of 4A51 (left), the ligand of 4BBG (right) and YL001 (below): positive charge (blue grid), negative charge (red grid). (**D**) Structure of STLC (left) and YL001 (right). (**E**) Docking pose of YL001 in the allosteric pocket of the receptor (PDB ID: 4A51). 2D interaction plot (left): hydrogen bonds (black dashes), pi-pi stacking interaction (green dashes). Surface plot (right): carbon (green), nitrogen (blue), oxygen (red), polar hydrogen (white).

The workflow for virtual screening is shown in Figure 1A. The co-crystallized conformation of five STLC analogues (including STLC) with strong bioactivity (PDB ID: 2XAE, 3KEN, 4A50, 4A51 and 4BBG) were extracted, aligned and utilized to generate queries for the similarity search. These queries were subsequently validated through ROCS using the Database of Useful Decoys Enhanced (DUD-E) Eg5 inhibitor subset. The best query (query5), which featured ligands from 3KEN, 4A50 and 4BBG, and the best ROCS scoring method (ranked by shape tanimoto only) were selected due to their extraordinary performance in validation results (Supplementary Figure 2B–2D, 2F). Parameters for molecule conformation generation (OMEGA, OpenEye) were optimized and single-molecule queries for EON were subsequently validated and selected. Ligands of 4A51 and 4BBG were selected as single-molecule queries for EON rescoring, as they both showed enhanced performance compared to the Non-EON group and random selection group (Supplementary Figure 2E). Molecules from the Specs database (204,000 compounds) were filtered with FILTER (OpenEye) to exclude molecules with an unfavorable druggability profile; prepared with OMEGA using optimized parameters; screened, scored and filtered through ROCS and EON; docked into the allosteric pockets of three individual Eg5 crystal structures (PDB ID: 4A50, 4A51 and 4BBG; Figure 1E) to inspect the calculated binding affinity and the binding mode; clustered by structure, and manually selected. In the virtual screening, YL001 showed good molecular shape similarity with query5, with a shape tanimoto score of 0.70 (Figure 1C). This molecule also ranked first in EON electrostatic shape comparison. In Figure 1D, it is evident that the N,N-dimethylamine group closely mimics the amino group from the query, while the trifluoromethyl group simulates the function of the alkyl group. Docking studies were conducted with AutoDockVina to determine whether the superpositioned conformation was consistent with the docking results [31]. For three different receptors (4A50, 4A51 and 4BBG) the average binding affinity (*n* = 3) for the best binding conformations of YL001 were −8.9, −9.4 and −9.2 kcal/mol respectively. A hydrogen bond was found between the protonated N,N-dimethylamine group and Glu116, and the trifluoromethyl group fitted into the subpocket where the alkyl group of the original ligands was located, adequately filling the pocket as in the superpositioned conformation. This validated the ROCS and EON results (Figure 1B, 1E). After completion of the workflow, 23 molecules were purchased from Specs for evaluation in further assays.

### Validation of YL001 as a highly selective antitumor agent targeted on Eg5

All 23 compounds selected by virtual screening were investigated with a novel comprehensive validation strategy to directly pick hits. This strategy combined enzymatic screening and SPR (as target-based screening) with cytotoxic and monopolar spindle screening (as phenotypic screening with high content imaging), allowing us to take advantage of both phenotypic and target-based screening, as well as to validate the compounds with strong anti-Eg5 activity (Supplementary Table 1). YL001 was selected using this strategy, and showed an EC_50_ of 1.18 μM on enzymatic assay, as well as an EC_50_ of 14.27 μM in HeLa cells with a monopolar spindle phenotype. Moreover, it bound to the Eg5 motor domain tightly, with a KD of 1.327×10^−7^ M as detected by SPR (Table 1). YL001 exhibited a KD constant which was two-fold stronger than the positive control STLC (3.767 × 10^−7^ M), and an order of magnitude stronger than compound 7170 which was identified in the first round of virtual screening (1.131 × 10^−6^ M). Through use of double validation with phenotypic and target-based screening, YL001 was identified as an Eg5 inhibitor with significant *in vitro* antitumor activity without obvious cytotoxicity against normal cells (Supplementary Table 2).

Activity and selectivity are two critical properties for small molecule enzyme inhibitors. Selectivity was a concern since YL001 has an α,β-unsaturated carbonyl bond which may react with endogenous nucleophiles via Michael addition and lead to cross-reaction with proteins *in vivo*. However, we found that YL001 is not active enough to react with highly active nucleophiles. YL001 was incubated with cysteine and lysine for 24 hours at 37°C and no new products were detected by HRMS. To further quantitatively analyze the selectivity of YL001, we assessed YL001 activity in a comprehensive panel of 468 typical, atypical and mutant human kinases at a concentration of 1 μM [32]. As expected, with a selectivity score of 0.00, YL001 displayed no activity at a concentration of 1 μM (taking into consideration the ~1% system false positive rate) (Supplementary Figure 3). These results confirm YL001 is not a covalent inhibitor, but a highly selective Eg5 inhibitor.

### YL001 induces a monopolar spindle phenotype, resulting in mitotic arrest and cell death

**Figure 2:**
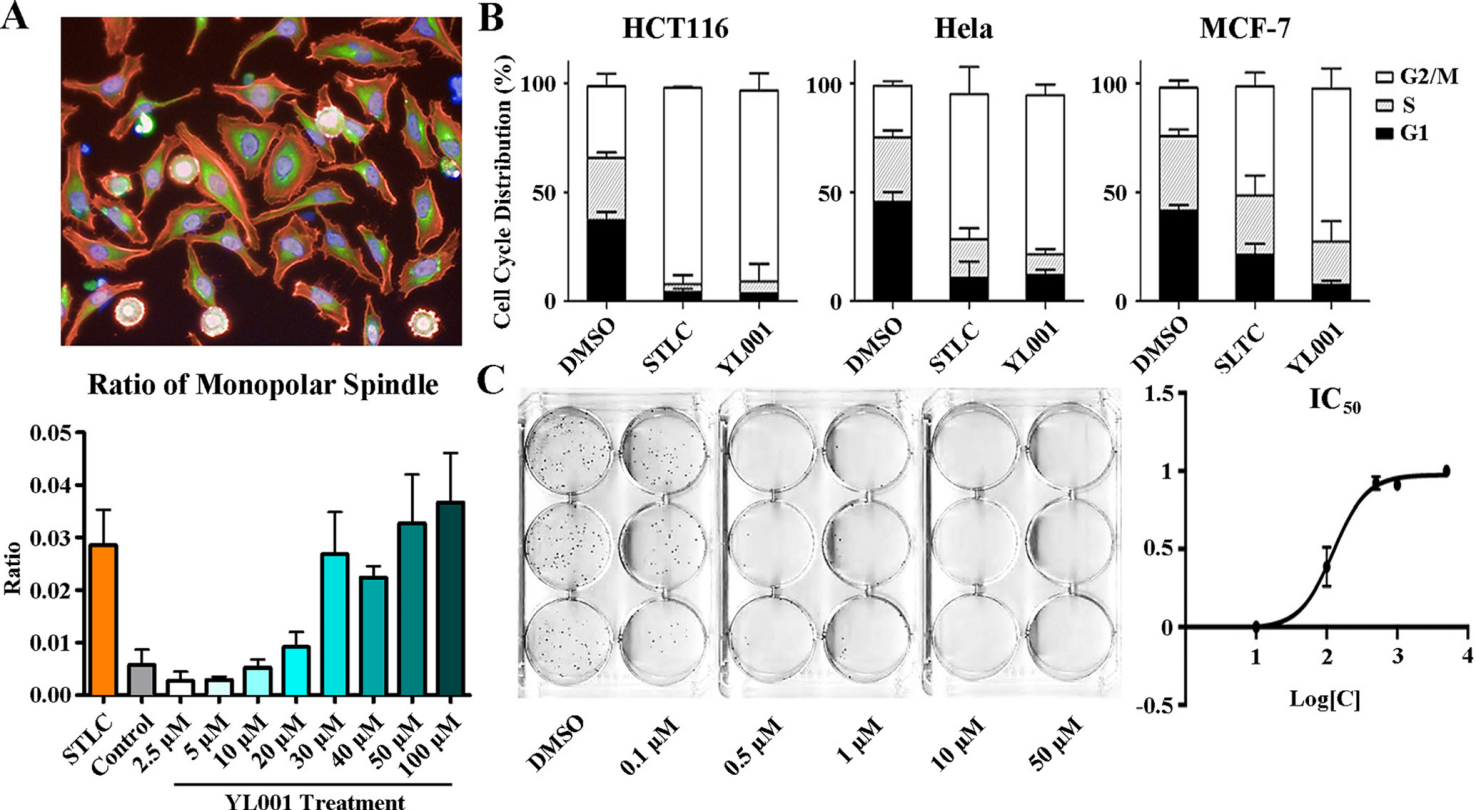
Phenotypic analysis of cells treated with YL001 (**A**) HeLa cells were treated with 50 μM YL001 or STLC (control) for 8 hours and fixed and stained for high content screening. Actin (red staining); tubulin (green staining); DNA (blue staining). The bar graph below shows the ratio of monopolar spindle cells to all cells treated with YL001 at different concentrations. (**B**) Cell cycle analysis of cells treated with YL001 in various cancer cell lines. YL001 efficiently arrested cells in mitosis and caused cell death. (**C**) HeLa cells were treated with gradient concentrations of YL001. The graph to the right shows inhibition with different treatment. YL001 exhibited antitumor activity by suppressing tumor growth in the colony formation assay.

YL001 strongly inhibited cell cycle progression. Exposure of cultured cells to YL001 produced the hallmark phenotype of Eg5 inhibition with an accumulation of cells in G2/M phase. High content imaging was performed on HeLa cells over a series of gradient concentrations of YL001, which induced a monopolar spindle phenotype in which cells showed a rosette of condensed chromosomes attached to a radial array of microtubules, wrapped in a round actin skeleton. As analyzed with Columbus, treatment with this compound led to monopolar spindles with an EC_50_ of 15.30 μM, demonstrating that normal mitotic spindle configuration is disrupted (Figure 2A).

Cytoflow was conducted on multiple cancer cell lines. As expected, all cell lines tested (HCT 116, HeLa and MCF7) showed arrest at G2/M, especially HCT 116. After 8 hours treatment with 50 μM YL001, the percentage of cells with 4N DNA content surged to over 80% (Figure 2B). To further observe the effect of YL001 induced cell cycle arrest, colony formation using different concentrations of the compound was carried out to measure the IC_50_. HeLa cells were exposed to YL001 for 24 hours and maintained in culture for 10 days without further treatment. The number of clones following YL001 treatment was significantly less than the number following STLC treatment. YL001 inhibited colony formation with an IC_50_ of 124 nM, demonstrating that in regard to antitumor activity, YL001 is stronger than STLC (IC_50_ = 1.25 μM) by an order of magnitude (Figure 2C).

### YL001 suppresses tumor growth and prolongs median survival by suppressing Eg5 function in a B16 melanoma xenograft model

YL001 activity was tested in multiple human and rodent cancer cell lines to evaluate its spectrum of antitumor activity. YL001 showed broad-spectrum antitumor effects with EC_50_ values predominantly in the range of 5–20 μM (Table 2). CNE-2Z (nasopharyngeal cancer) and K562 (leukemia) cells were especially sensitive to treatment. HT1080 with an EC_50_ of over 70 μM was the only cell line which was not sensitive to treatment among the 15 cell lines tested. According to BioGPS data [33], in the four cell lines with available Eg5 gene expression level data, Eg5 expression ranked as HeLa > MCF-7 > U87 > HT1080. This shows the efficiency of YL001 activity is positively correlated with Eg5 gene expression. In addition, Eg5 inhibitors would be expected to be effective in cancers which are resistant to tubulin-targeting agents [34], where resistance results from mutation of beta-tubulin or alteration in the expression of tubulin isoforms [35, 36]. As expected, YL001 has shown activity in the taxol resistant cell line A2780 and the 6-Thiaguanine resistant cell line 4T1, which suggests YL001 has significant potential for therapy of drug-resistant tumors.

**Table 2:** Anti-proliferative activity EC_50_s of YL001 against a broad range of human and rodent cancer cell lines

Cell Lines	Organism	Type	Source	EC_50_ of YL001/ μM
K562	Human	Lymphoid	Leukemia	9.76 ± 0.52
CNE-2Z	Human	Epithelial	Nasopharyngeal cancer	5.94 ± 0.02
A549	Human	Epithelial	Lung cancer	22.77 ± 0.72
Huh7	Human	Epithelial	Liver cancer	14.59 ± 0.82
HeLa	Human	Epithelial	Cervical cancer	14.27 ± 0.78
B16	Mouse	Epithelial	Melanoma	13.91 ± 5.03
HCT116	Human	Epithelial	Colon cancer	9.04 ± 2.19
U87	Human	Epithelial	Brain cancer	26.20 ± 0.12
DU145	Human	Epithelial	Prostate cancer	26.46 ± 0.27
MCF-7	Human	Epithelial	Breast cancer	14.90 ± 2.57
786-O	Human	Epithelial	Renal cancer	14.69 ± 0.62
AGS	Human	Epithelial	Stomach cancer	20.20 ± 0.01
HT1080	Human	Epithelial	Fibrosarcoma	76.25 ± 0.77
A2780/taxol	Human	Epithelial	Ovarian cancer	20.14 ± 0.68
4T1/6TG	Mouse	Epithelial	Breast cancer	20.03 ± 0.50

Thirteen human cancer cell lines and two rodent cancer cell lines were used, including a human resistant cell line and a mouse resistant cell line. A2780/taxol is a taxol resistant A2780 cell line and 4T1/6TG is a 6TG resistant 4T1 cell line. Cellular activity of these three compounds showed positive correlation with Eg5 gene expression (HeLa > MCF-7 > U87 > HT1080).

**Figure 3:**
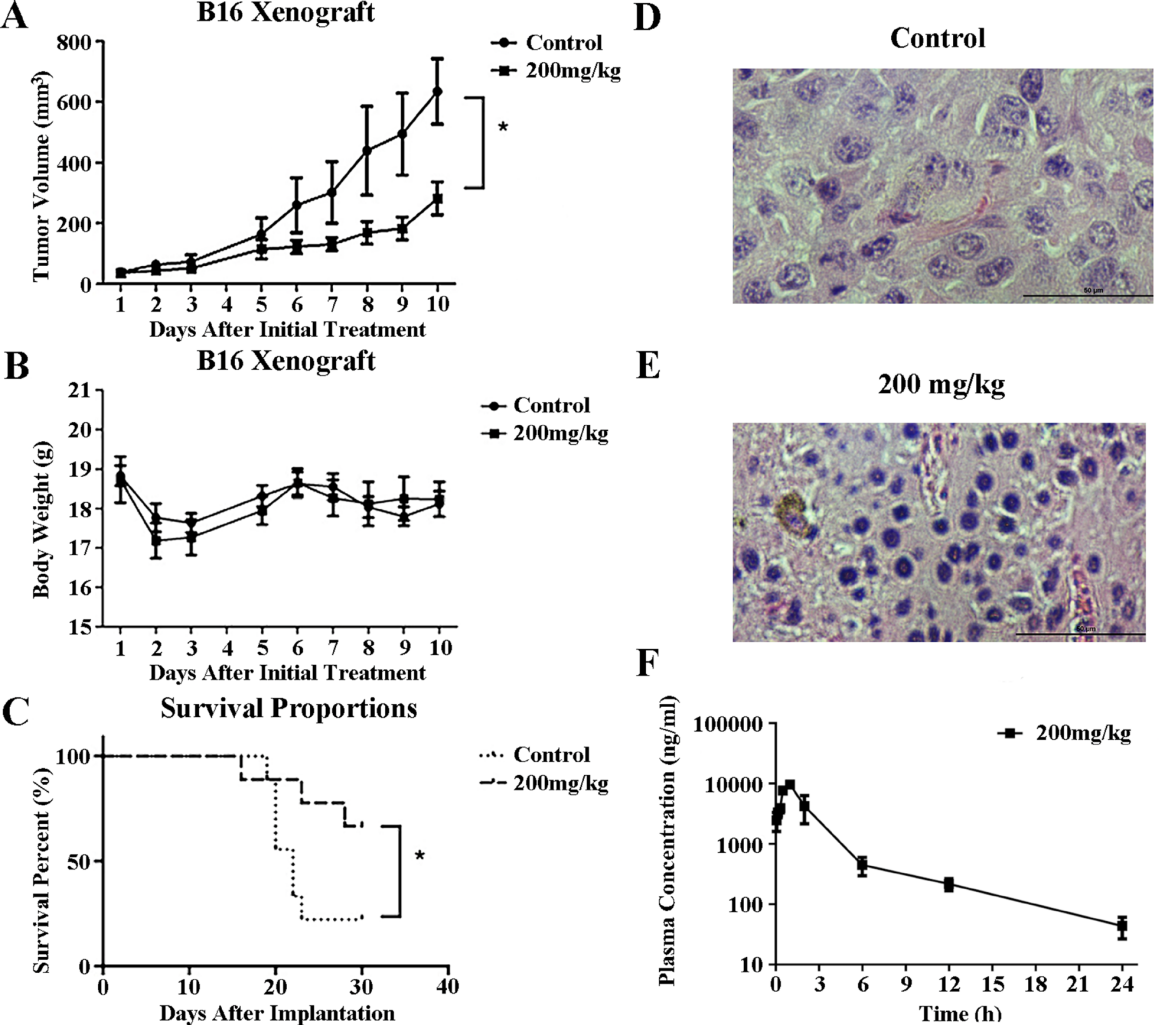
Antitumor efficacy of YL001 in a xenograft tumor model (**A**) Tumor volume versus time after initial treatment with YL001 in B16 tumor-bearing mice. Columns, mean (*n* = 6); bars, SD. *P < 0.05. (**B**) Body weight versus days after initial treatment with YL001 in B16 tumor-bearing mice. Columns, mean (*n* = 6); bars, SD. (**C**) Percent survival versus time after implantation in B16 tumor-bearing mice treated with YL001 (*n* = 9). *P < 0.05. (**D**) H&E staining of a tumor from the control group. (**E**) H&E staining of a tumor from the YL001 treated group. (**F**) Pharmacokinetic study of YL001. Plasma concentration versus time after initial administration with YL001 in HeLa tumor-bearing Balb/c nude mice. Columns, mean (*n* = 3); bars, SD.

The strong broad-spectrum antitumor activity displayed by YL001 against cultured cells prompted further evaluation of *in vivo* activity of YL001 in a B16 rodent melanoma xenograft model. After testing a range of YL001 doses in healthy B6 mice without tumor, we estimated the maximal therapeutic dose to be 200 mg/kg taking into consideration the solubility of YL001. Doses of 200 mg/kg were administrated daily for 10 days to B6 mice with tumor xenografts of the highly malignant melanoma B16. Animals with this xenograft in general exhibit a low survival rate and poor response to chemotherapy. However, *i.p*. treatment with YL001 significantly suppressed tumor growth with a minimum T/C ratio of 0.40 (*P* < 0.05 for tumor volume compared to controls) (Figure 3A) and an absence of toxicity (*P* > 0.05 for body weight loss compared to controls) (Figure 3B). Median survival results (Figure 3C) showed prolongation of the treatment group's survival time by 50% over that of the control (Table 3). YL001 thus has excellent antitumor activity in this xenograft model, and no adverse effects were observed.

**Table 3:** Pharmacokinetic/pharmacodynamic studies of YL001

	Control	YL001 200 mg/kg
Medium survival time/day	22	> 33
Half-life in plasma/hour	–	5.3

Median survival time is shown in days. Half-life in plasma is presented in hours.

To determine whether YL001 inhibited Eg5 function in the B16 xenograft model, we examined the phenotype of mitotic cells in tumor tissues. Histochemisty analysis of tumor tissue demonstrated that YL001 induces mitotic arrest accompanied by formation of monopolar spindles through inhibition of Eg5 (Figure 3D, 3E), and has antitumor activity in xenograft tumor models (Figure 3A, 3C).

We also assessed the half-life of YL001 in the plasma in HeLa xenograft nude mice. The results (Figure 3F) show YL001 has a favorable pharmacokinetic property with t_1/2_ = 5.3 hours. These results indicate that YL001 has favorable pharmacokinetic and pharmacodynamic characteristics which warrant further investigation of this agent as an antitumor agent.

### YL001 causes mitotic failure leading to cell death through activation of caspase-3

High content imaging in the YL001 treated group of cells showed cell fragments including many apoptotic bodies reflecting apoptosis, which leads to cell death by the activation of caspases. Caspase-3 is the most important of these caspases and plays a central role in transduction of apoptotic signals as an effector, and its activation leads to protein degradation.

**Figure 4:**
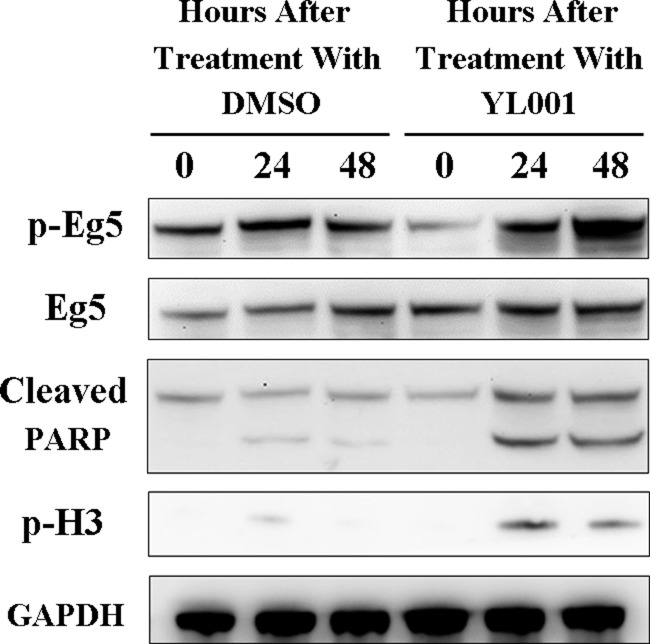
YL001 triggers caspase-3 activation and elevates Eg5 phosphorylation at T926 HeLa cells were treated with 50 μM of YL001 and DMSO (as a control) for a given length of time and cell lysate was tested for T926 phosphorylation of Eg5, total Eg5, cleaved PARP, S10 phosphorylation of histone H3 and GAPDH (control).

Western blot was used for analysis of cleaved PARP1, which is a product of Caspase-3 that serves as a marker for intrinsic apoptosis. After 24 hours treatment with YL001, PARP1 cleavage increased dramatically (Figure 4A, lane 2 vs. lane 5), and the accumulation of cleaved PARP1 persisted for 48 hours (Figure 4A, lane 3 vs. lane 6). This shows YL001-induced cell death is due to apoptosis caused by Caspase-3 activation [34]. In addition, the marker of phosphor histone H3, which is specifically phosphorylated in mitosis, was evaluated with western blot. Immunostaining with phospho-specific antibody revealed that with YL001 treatment, the cells are evidently arrested in mitosis (Figure 4A, lane 5–6), leading eventually to apoptosis.

**Figure:**
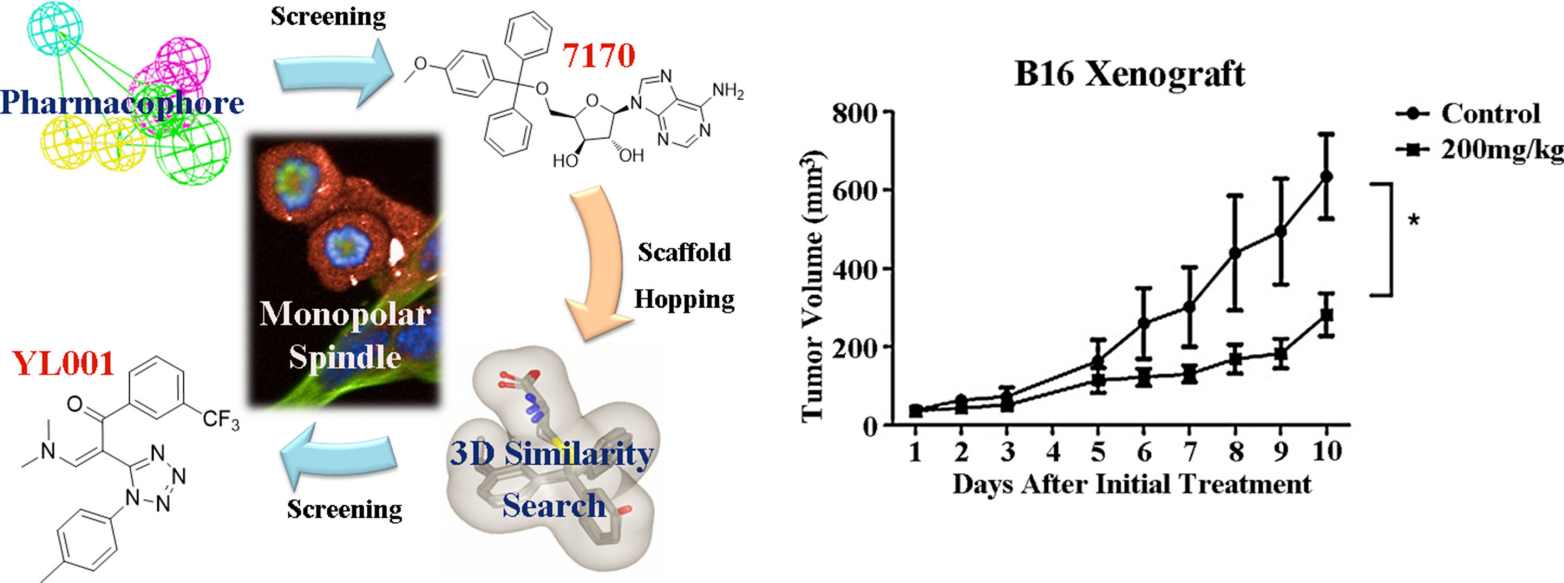
Graphical Abstract: Discovery of Eg5 inhibitor YL001 as a novel antitumor agent Left, two rounds of virtual screening results in the discovery of YL001 as an Eg5 inhibitor which can lead to monopolar spindle phenotype. Right, YL001 exhibits potent antitumor activity in the B16 xenograft mouse model.

It came as a surprise to find comparison of each specific time point showed YL001 treatment consistently elevated phosphorylation of Eg5 at T926 (Figure 4A, lane 2–3 vs. lane 5–6). Eg5 phosphorylation is essential for its functional association with microtubules [2, 37, 38], but Eg5 hyperphosphorylation impairs normal Eg5 distribution and its function in spindle assembly [39]. These results demonstrate YL001 not only impedes Eg5 ATPase function as shown in Result 2.2, but also induces Eg5 hyperphosphorylation at T926, leading to mitotic catastrophe and subsequent apoptosis.

## DISCUSSION

In this study, we have successfully combined virtual screening, target-based screening and phenotypic screening as a novel strategy to discover a novel scaffold Eg5 inhibitor with strong anti-neoplastic activity economically and efficiently. This novel strategy may offer guidance for discovery of other new targeted drugs.

Query is the core of a 3D similarity search in virtual screening. STLC was a structurally simple lead compound with distinctive antitumor activity [19, 20]. As numerous previous studies had discussed its SAR and co-crystallization structures with Eg5 [40–43], it was clear that STLC analogues were suitable for building our query, and supporting effective screening for novel modulators of the target as well. Analysis of co-crystal structures of STLC with Eg5 showed that the crystallized bioactive conformation of these STLC analogues superimposed well within the Eg5 allosteric pocket. We therefore supposed that use of a highly congruent superimposed bioactive conformation as a shape query would not only provide greater space for accommodating the molecular overlay, but would also circumvent the limitations of single molecule queries, and thus would be more compatible with discovery of new scaffolds.

Over the course of development of this compound, its chemical scaffold underwent a substantial structural change from the triphenylmethyl scaffold (STLC) to the final novel 1,5-disubstituted tetrazole (YL001). This would have been difficult to achieve using conventional drug design approaches such as bioisosteric fragment replacement. However, this virtual screening workflow yielded successful scaffold hopping. Similarity search based methodology generally outperforms molecular docking in efficiency, and 3D-similarity search achieves scaffold hopping albeit at the cost of a decrease in the success rate when compared to traditional 2D-similarity searches and molecular docking [44–46]. Thus, considering the small pool size of the compounds tested, the success rate (one hit out of 23 molecules tested, which is 4.3%) was satisfactory.

In phenotypic screening, an effective high content imaging method was introduced to identify potential hit compounds. In current practice, screening based on phenotype plays a more critical role in the early stages of drug discovery. Prior to the 1980s, most drugs were discovered with use of phenotypic screening *in vitro* or *in vivo*. However with the development of clone technology, phenotypic screening in drug discovery was gradually de-emphasized under comparison with the then emerging high-throughput, target-specific *in vitro* assays [47]. During the heyday of targeted cancer therapy at the beginning of 21^st^ century, numerous potential drug targets were discovered, and screening assays were constructed based on these targets. However, this approach yielded many clinical candidates but resulted in few new marketed drugs [48]. This was attributed to the fact that target-based drug screening did not take disease pathogenesis into account, and targeted drugs still incur the risk of inducing adaptive drug resistance [49]. We surmised that by combining phenotypic and target-based screening, advantages of both methods could be maximized and disadvantages could be minimized. This provided a novel platform for drug discovery which is economical and effective, and is adaptable to other research systems.

As expected, introduction of YL001 resulted in failure of mitosis by inhibiting ATPase function and accumulation of T926 phosphorylated Eg5, thus facilitating apoptosis by activating caspase-3. Although the mechanism of its activity has not been fully worked out, our results are sufficient to show YL001 has promise as an Eg5 inhibitor for cancer therapy.

## MATERIALS AND METHODS

### Virtual screening

#### Virtual screening round one procedure

A HypoGen pharmacophore model was generated by Discovery Studio 2.5 modeled on the study of Liu et al. [27]. Compounds from the PKU database were prepared and screened through this pharmacophore model, which contains four features including an aromatic ring, a hydrophobic, a hydrogen bond acceptor and a donor. The Eg5-ispinesib complex crystal structure (PDB code 4AP0) was used for *in silico* docking via GOLD 3.0. All compounds tested interacted with the Eg5 allosteric pocket. 100 top scored compounds in both two models were kept in the best-binding conformations for virtual analysis. Eventually six low weight molecules were obtained for the further validation.

### Virtual screening round two procedures

#### Query validation and parameter optimization with OMEGA, ROCS and EON

Five STLC-typed ligands with strong binding capacity in their crystalized and bioactive conformations were selected (PDB ID: 2XAE, 3KEN, 4A50, 4A51 and 4BBG). The ligand 4BBG was modified to remove the unwanted aromatic tautomer state, and was then aligned with Discovery Studio V2.5 (Accelrys) using the default settings of the molecular overlay module. Five Queries were generated using aligned molecules by vROCS 3.1.2 (OpenEye) with the following parameters: maximum molecules per model = 5, models to keep = 5, merge color atoms = True. Four of five output queries were multi-ligand queries, and one query was a single-compound query. ROCS query validation was validated with the KIF11 subset of DUD-E selected to assess the performance of different queries, scoring functions and running modes [50]. Conformations of the validation set were generated by OMEGA 2.4.6 (OpenEye) using default parameters with the FLIPPER module turned on. All of the single-compound queries acquired directly from the original crystalized conformations were assessed as well. Query validation data is recorded in Supplementary Figure 1. Parameters of OMEGA were also subsequently optimized on query5 using the same validation method. Ranges of all relevant parameters (“ewindow” from 5, 10 to 15 kcal/mol and “rms” from 0.3, 0.4, 0.5 to 0.6 angstroms respectively) were chosen according to several important references. For maximum efficiency, we had the parameter “maxconfs” set to 100. The top 1% of the ROCS output hit list (ranked by shape tanimoto) for the optimized method was used as an input for EON (2.1.0, OpenEye) query validation. Since EON only accepts single-molecule queries, only the single-compound queries acquired directly from the original crystalized conformation were evaluated using the default parameters. The output was ranked by ET_Combo (Supplementary Figure 1F).

### Database preparations

The Specs database (~204,000 compounds) was used as the screening library. All compounds were first treated with Pipeline Pilot v7.5 (Accelrys) to generate 3D coordination, strip salt, minimize molecule energy, and standardize the chemical table coding. FILTER 2.1.1 from OpenEye was then applied to filter unfavorable compounds using the recommended filter criteria documented in filter_blockbuster.txt provided by OpenEye. Conformations were generated by OMEGA 2.4.6 using the optimized parameters with the FLIPPER module turned on.

### Virtual screening

All conformations were compared to query5 using ROCS 3.1.2 on molecular shape and ranked by shape tanimoto. The first 5,000 molecules were kept and re-evaluated by EON 2.1.0 to preserve the first 500 molecules with the highest ET_Combo score. Two separate hit lists produced by using the two different EON queries were merged with Pipeline Pilot. Molecules with the following properties were discarded: ADMET Solubility Level = 0, rotatable bonds > 9 (both properties calculated by Pipeline Pilot), which left 389 molecules for post screening analysis.

### Pose screening analysis

All 389 molecules were docked into the allosteric pocket of three different receptors (PDB ID: 4A50, 4A51 and 4BBG) by AutoDockVina (1.1.2, Scripps) with default settings. Best-binding conformations were manually inspected in Open-Source PyMOL v1.3.x (Schrödinger). Hydrogen bond and ligand embedding ratios were speculatively assigned and calculated by the third-party PyMOL scripts. In the three separate docked poses corresponding to each receptor, at least two were expected to be successfully docked into the allosteric pocket to be considered a reliable pose, and the average embedding ratio of those poses was expected to be larger than 0.6. Moreover, at least two reliable poses were expected to have no less than one hydrogen bond interaction with the receptor, and the average hydrogen bond interaction count of these reliable poses was larger than one. In addition, the average binding affinity for these reliable poses was expected to be stronger than -9 kcal/mol. After docking analysis, 77 molecules were kept for further study. These molecules were clustered using the Ligand Cluster protocol provided by Pipeline Pilot, and the results were modified manually for better results. No more than two molecules were selected per cluster. Finally, 24 molecules were confirmed, and 23 of these were purchased from Specs (one of them was unavailable) to be evaluated in further assays. The 2D binding plot was generated by the PoseView web service [51].

### Organic synthesis

Solvents and reagents were obtained from commercial suppliers and were used without further purification. Reactions were monitored with thin layer chromatography (TLC) on GF254 silica gel plates (Anhui LiangChen Silicon Material Co. Ltd.). The spots were visualized using an ultraviolet light. Flash chromatography was carried out on silica gel zcx-II (48–75 μm, Branch of Qingdao Haiyang Chemical Co. Ltd.) using the indicated solvents (PE, boiling range 60–90°C). ^1^H NMR spectra and ^13^C NMR spectra were recorded on a Bruker AVANCE III 400 (400 MHz) spectrometer in D-reagents with tetramethylsilane (TMS) as an internal reference. NMR chemical shifts are reported as values (ppm) relative to internal TMS. Splitting patterns were designated as: s = singlet, d = doublet, t = triplet, q = quartet, m = multiplet. Melting points were measured as uncorrected values. High resolution mass spectrometry (HRMS) was measured with an LTQ ObitrapElite spectrometer using ionspray methodology. High-performance liquid chromatography (HPLC) was carried out using a ZORBAX SB-C18 column 5 μm, 4.6 × 250mm.

Typical synthesis procedure for the target compound YL001 ((Z)-3-(dimethylamino)-2-(1-(p-tolyl)-1H-tetrazol-5-yl)-1-(3-(trifluoromethyl)phenyl)prop-2-en-1-one)

Step 1: A solution of 4.0 mL (30 mmol, 1 eq.) 4›-methyl-acetophenone was added to N,N-Dimethylformamide dimethyl acetal 8.0 mL (60 mmol, 2 eq.) and this mixture was stirred at 115°C under reflux for 30 hours. The reaction mixture was then cooled to room temperature, and red crystals were precipitated. The product was collected through suction filtration, washed with PE (15 mL × 3) and dried in vacuo. The product (4.532 g, 23.9 mmol), consisting of orange crystals, was pure enough to be used directly in the next reaction. The yield was 79.7%.

Step 2: To a solution of the product from step 1 (3.730 g, 19.7 mmol, 1 eq.) in 13.1 mL cold anhydrous dichloromethane (DCM) stirred at 0°C, a solution of POCl_3_ (1.84 mL, 19.7 mmol, 1 eq.) in 4.9 mL cold anhydrous DCM was added dropwise. This mixture was then stirred at room temperature for 20 minutes and 0°C for another 10 minutes. The intermediate was collected through suction filtration, and washed with 10 mL cold anhydrous DCM. While filtering the intermediate, 8.276 g (60.4 mmol, 3.1 eq.) of NaClO_4_·H_2_O was dissolved in 16.4 mL water and stirred at 0°C. The intermediate was added to this solution and stirred vigorously at 0°Cfor 30 minutes. The product was then collected by suction filtration, washed with a solution of 1.642 g (11.7 mmol, 0.6 eq.) NaClO_4_·H_2_O in 16.4 mL water, and dried in vacuo. The product (6.795 g) was as light yellow solid, was pure enough to be used directly in the next reaction. Yield was considered as 100%.

Step 3: In a bottle with two necks 2.945 g (45.3 mmol, 2.3 eq.) of NaN_3_ and 50 mL methanol were stirred at 60°C under reflux. Product from step 2 was added portion wise, and gas was allowed to release completely before adding the next portion. After product from step 2 was completely added, the mixture was stirred for another 30 minutes at 60°C under reflux. The mixture was then cooled to room temperature. Excess distilled water was poured into the mixture while stirring to let the product precipitate. The product was filtered by suction filtration, washed with diluted water (10 mL × 2), and dried in vacuo. The product (2.300 g, 10 mmol) was as pink solid, and was pure enough to be used directly in a next reaction. Yield was 50.5%.

Step 4: To a solution of the product from step 3 (229 mg, 1.0 mmol, 1 eq.) in 5 mL anhydrous toluene, triethylamine 0.17 mL (1.2 mmol, 1.2 eq.) was added and stirred at 0°C for 10 minutes. 3-(Trifluoromethyl)benzoyl chloride (1.2 mmol, 1.2 eq.) was added dropwise, and stirred for another 30 minutes at 0°C. The mixture was stirred and heated to 105°C under reflux for 8 hours. The mixture was then cooled to room temperature, and 10 mL EA and 50 mL of water were added to the mixture and extracted. The water phase was extracted with EA (10 mL × 3), and the organic phases were combined and washed with brine (50 mL × 3). The organic phase was dried with anhydrous Na_2_SO_4_. After the solvent was removed under vacuum, the residue was separated with silica gel column chromatography (PE: EA = 1: 1 to 1: 2 to 100% EA) which yielded the crude product. The crude product was heated and recrystallized in methanol, the crystal product was washed with anhydrous ether three times, and dried in vacuo. Final product, YL001, was as light yellow crystal, mp. 122.2 - 122.4°C. ^1^H NMR (400 MHz, CDCl_3_) δ 7.90 (s, 1H), 7.52 (d, J = 7.9 Hz, 1H), 7.22 (t, J = 7.8 Hz, 1H), 7.07 (m, 4H), 6.91 (d, J = 7.9 Hz, 2H), 3.34 (s, 3H), 2.75 (s, 3H), 2.36 (s, 3H). ^13^C NMR (100 MHz, CDCl_3_) δ 189.09, 156.83, 151.38, 140.26, 139.88, 131.62, 130.95, 130.63, 130.03, 128.22, 126.86, 125.04, 124.86, 123.51, 122.76, 122.15, 91.34, 77.37, 77.05, 76.73, 48.01, 40.87, 21.01. HRMS calcd. for C_20_H_18_F_3_N_5_O^+^ [M+H]^+^ 402.15362, found 402.15337, ppm 1.96.

### Biology

#### Cancer cell lines

Cancer cell lines (CNE-2Z, A549, Huh7, HeLa, B16, K562, MCF-7, HCT116, U87, DU145, 786-O, AGS, HT1080, A2780/taxol, 4T1/6TG) used for both *in vitro* antitumor and mechanism studies, and the *in vivo* xenograft mouse model were all obtained from the China Infrastructure of Cell Line Resources and American Type Culture Collection. Cells were cultured in DMEM (Macgene) or RPMI 1640 (Macgene) medium supplemented with 10% FBS in a 37°C incubator with 5% (v/v) CO_2_.

### Compounds and reagents

STLC was from Alladin (1121390) (Shanghai, China). The compounds of interest identified by virtual screening were purchased from Specs. YL001 was first purchased from Specs, then synthesized in our laboratory. All compounds were prepared as 10 mM DMSO stock, and were sequentially diluted in growth medium. Cells were treated with compounds by medium exchange.

### Antibodies

Cell lysates from cultured cells were prepared and analyzed by immunoblotting on nitrocellulose membranes. The following antibodies were used in this study: Mouse total anti-Eg5 T926 (620502) anti-Eg5 total (627802) was purchased from Biolegend. Rabbit anti-cleaved PARP (D64E10) (CS-5625P) and anti phospho histone H3 (ser10) (CST3377) antibody was from Cell Signaling Technologies. Mouse anti-GAPDH antibody was from Zhongshan Golden Bridge Biotechnology (Beijing, China). Anti-rabbit IgG and anti-mouse IgG coupled horseradish peroxidase (HRP) were from Sigma-Aldrich (St. Louis, MO, USA).

### Inhibition of Eg5 ATPase Activity

All experiments were performed as previously described [52]. Data were analyzed using GraphPad Prism 5 and Microsoft Excel to obtain EC_50_ values. STLC was used as a positive control. Each inhibitory concentration was measured in triplicate, and averaged data points ± SD are shown.

### SPR Assay

Interactions of the Eg5 motor domain and compounds of interest were analyzed using the Biacore T200 system (GE Healthcare, Uppsala, Sweden) at 25°C. Eg5 motor protein obtained as previously described (51) was immobilized on a sensor chip (CM5) using an amine coupling kit (GE Healthcare, Buckinghamshire, UK). Final Eg5 immobilization levels were typically ~23000 RU. Subsequently, compounds were injected as analytes at various concentrations, using PBS-P (10 mM phosphate buffer with 2.7 mM KCl and 137 mM NaCl, 0.05% Surfactant P20, pH 4.5, GE Healthcare, Uppsala, Sweden) as a running buffer. For binding studies, analytes were applied at corresponding concentrations in running buffer at a flow rate of 30 μL/minute with a contact time of 60 seconds and a dissociation time of 60 seconds. Chip platforms were washed with running buffer and 50% DMSO. Data were analyzed with Biacore evaluation software (T200 Version 1.0) by curve fitting using a steady state 1:1 binding model and a kinetic model.

### Nucleophilic addition assay

0.5 mmol YL001 was solved in a solution of THF/H_2_O (v:v = 1:1) and stirred with nucleophiles (0.5 mmol L-cysteine or L-lysine) at 37°C for 24 hours. Products were then validated by HRMS.

### Kinase profiling

Kinase profiling was conducted by the DiscoverX as KINOMEscan profiling service for testing the inhibition of YL001 of interest against 468 kinases [53]. Selectivity score S(35) was defined as follows:

S(35) = (number of non-mutant kinases with% Control < 35)/(number of non-mutant kinases tested).

### Antiproliferation assays

Cytotoxic activities of the compounds of interest were investigated with human cancer cell lines using the Alamar Blue assay. Cells (5 × 10^3^ cells/well) were seeded into 96-well plates (Corning, 3603). Various sample concentrations (compounds of interest) were added to each well in duplicate, and incubated at 37°C with 5% CO_2_ for two days such that cells were in the exponential growth phase at the time of drug addition. Ten microliters of the Alamar Blue Solution (Invitrogen, Alamar Blue) were added to each well and incubated at 37°C for up to 2 hours in a humidified, 5% CO_2_ atmosphere. After incubation, florescence was measured using a microplate reader (Tecan) at an excitation wavelength of 540 nm and emission of 590 nm. Each inhibitory concentration was measured in triplicate, and averaged data points ± SD are shown. Statistical analysis was carried out with GraphPad Prism 5 and the fifty-percent effective concentration is expressed as EC_50_.

### Immunofluorescence microscopy

HeLa cells (5 × 10^3^ cells/well) were seeded in 96-well plates (Corning, 3603). Drugs were diluted appropriately in medium from 10 mM stocks in 100% DMSO and added to the cells. Following 8 hours incubation with drugs, cells were fixed with 4% paraformaldehyde (20 minutes at room temperature), permeabilized with PBST (0.5% Triton X-100 in PBS) for 15 minutes, and washed before blocking. Cells were blocked with 4% BSA for at least 30 minutes at room temperature and stained with anti-β-tubulin monoclonal antibodies (Sigma) at 400-fold dilution for 1 hour with Alexafluor 488-conjugated goat anti-mouse secondary antibodies (Invitrogen) at 300-fold dilution for 1 hour. DNA was detected with 4′,6-diamidino-2-phenylindole dihydrochloride (DAPI). Actin was detected with Phalloidin. High content screening (Perkin Elmer Operetta) was used to photograph stained cells at 40× magnification, and Columbus software was used for statistical analysis of the percentage of mitotic cells with monopolar spindles present in treated cells over the total number of cells in mitosis counted after 8 hours incubation with drugs.

### Flow cytometry

HeLa cells (1 × 10^6^ cells per well) were plated in 6-well plates (Nunc, 140675). 10 mM stock was diluted into an appropriate final concentration solution with growth medium. After 12 hours, cells were incubated with compounds for 12 hours in 37°C with 5% CO_2_, using DMSO as a negative control and 50 μM STLC as a positive control. Medium was removed and PBS was used for washing twice. Cells were then digested with trypsin into a single-cell solution and fixed with methanol overnight at 4°C. Fixative was removed and cells were washed twice with PBS. 25 μL 20×RNaseA was added followed by incubation for 30 minutes at 37°C. 5 μL of PI was added followed by incubation for 30 minutes at 4°C. Cytoflow was carried out by BD FACSVerse (BD Biosciences, US).

### Colony formation

HeLa cells (5 × 10^2^ cells/well) were seeded into 6-well plates and were treated with a series of gradient concentrations of YL001 for 24 hours, followed by washing and continual culture for 10 days. DMSO was used as a negative control, and STLC was used as a positive control. Cells were fixed with ethanol for more than 30 minutes and dyed with crystal violet for more than 30 minutes. Clones were counted with OpenCFU [54].

### *In vivo* tumor xenograft studies

B16 cells (1 × 10^6^ cells) were inoculated *s.c*. into 6-week old C57BL/6 mice. Tumor volume was calculated as: V = 1/2× L (long axis)×W^2^ (short axis). When the volume of tumor was between 50 to 100 mm^3^, YL001 was administered *i.p*. daily on days 1 to 10 at 200 mg/kg. Doses and schedules were determined by tolerance studies. Mice were kept under specific pathogen-free conditions, and all animal experiments were approved by the Committee on Animal Research at our institute.

Vehicle (10% (v%) N,N-Dimethylacetamide (DMAC) in 200 μL olive oil per 20 g mouse) was administered daily *i.p*. as a control on days 1 to 10. Drug efficiency was expressed as the ratio of the mean experimental V/V_0_ value to that of the control group [treated versus control (T/C) ratio], where V is the tumor volume on the day of evaluation and V_0_ is the tumor volume on the day of initial treatment with the drug. Drug efficiency was also evaluated by median survival. All statistical analysis was performed using Graphpad Prism 5.

### Histochemistry

On day 10 after the first treatment, all mice were sacrificed. Tumors from two mice in each group were fixed with formalin and analyzed histologically using H&E staining.

### Pharmacokinetic study

A HPLC-MS/MS method was developed for single-dose pharmacokinetic (PK) study of YL001 in HeLa xenograft BALB/c nude mice. YL001 was administered intraperitoneally at a dose of 200 mg/kg. Blood samples were then collected by enucleating eyeballs at 0.083, 0.167, 0.333, 0.5, 1, 2, 6, 12, 24 hours in heparinized polypropylene tubes. Three mice were sacrificed at each time point. Samples were centrifuged immediately at 4000 r/min for 20 min to separate plasma. The final sample for HPLC-MS/MS detection by QTRAP 6500 (AB Sciex, USA) was a mixture (plasma: H_2_O: methanol = 1:1:8). All individual data were pooled together to further perform PK analysis. DAS 2.1.2 were used to estimate PK parameters by non-compartmental analysis.

## SUPPLEMENTARY FIGURES AND TABLES



## Abbreviations

KSP: Kinesin Spindle Protein
SAR: Structure-Activity Relationship
CADD: computer aided drug design
STLC: S-Trityl-L-cysteine
DUD-E: Database of Useful Decoys: Enhanced
SPR: Surface Plasmon Resonance
KD: Dissociation Constant
TLC: Thin Layer Chromatography
PPI: Protein-protein interaction
HRMS: High Resolution Mass Spectrometry
HPLC: High-Performance Liquid Chromatography
H&E: Hematoxylin and eosin
DMAC: N,N-Dimethylacetamide
