# Supplementary material for: Eg5 inhibitor YL001 induces mitotic arrest and inhibits tumor proliferation
Source: Oncotarget. 2017 Apr 18;8(26):42510–24. doi: 10.18632/oncotarget.17207 (PMC5522084; doi:10.18632/oncotarget.17207)
Supplement: Supplementary file 1 [file oncotarget-08-42510-s001.pdf]

Eg5 inhibitor YL001 induces mitotic arrest and inhibits tumor proliferation

Supplementary Materials

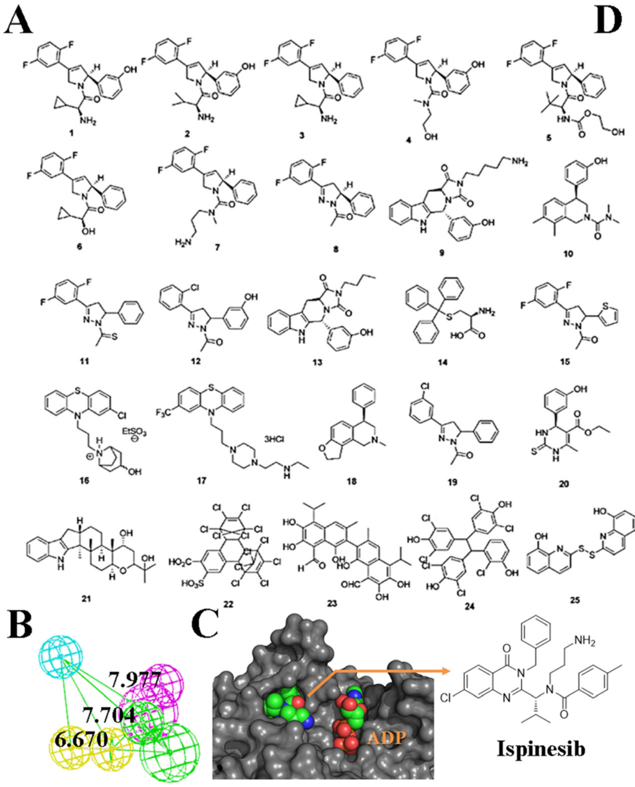

D

No.	ID Number	Structure	Binding Level (RU)	EC50 (μM)
6829	10005325	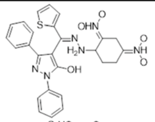	-0.6	NT
2627	100056372	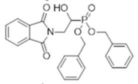	74.5	NT
7245	100056634	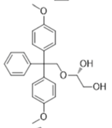	43.5	NT
7170	100056766	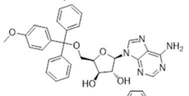	164.6	13.32
8178	100061411	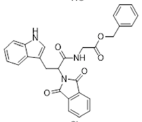	98.1	14.49
8397	100061289	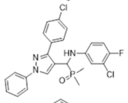	243.4	14.59
STLC		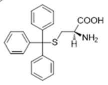	97.7	1.46

Supplementary Figure 1: Develop Eg5 inhibitors via receptor-ligand interaction-based virtual screening round one. (**A**) 2D chemical structures of the 28 molecules forming the training set used to obtain the HypoGen pharmacophore hypothesis. (**B**) Top-scoring HypoGen pharmacophore Hypo 1. Features are color-coded as follows: aromatic ring (orange); hydrogen-bond acceptor (green); hydrophobic (blue); hydrogen-bond donor (violet). (**C**) All compounds in the database tested interacted with the allosteric pocket which is occupied by Ispinesib in the picture (GOLD). PDB ID: 4AP0. (**D**) Low weight molecule screening of the six manually selected compounds: Binding level (Response Unit, RU) was evaluated with SPR Biacore T200 and EC_{50} was assessed with the Alamar Blue assay in cervical cancer cell line HeLa. STLC was used as a control.

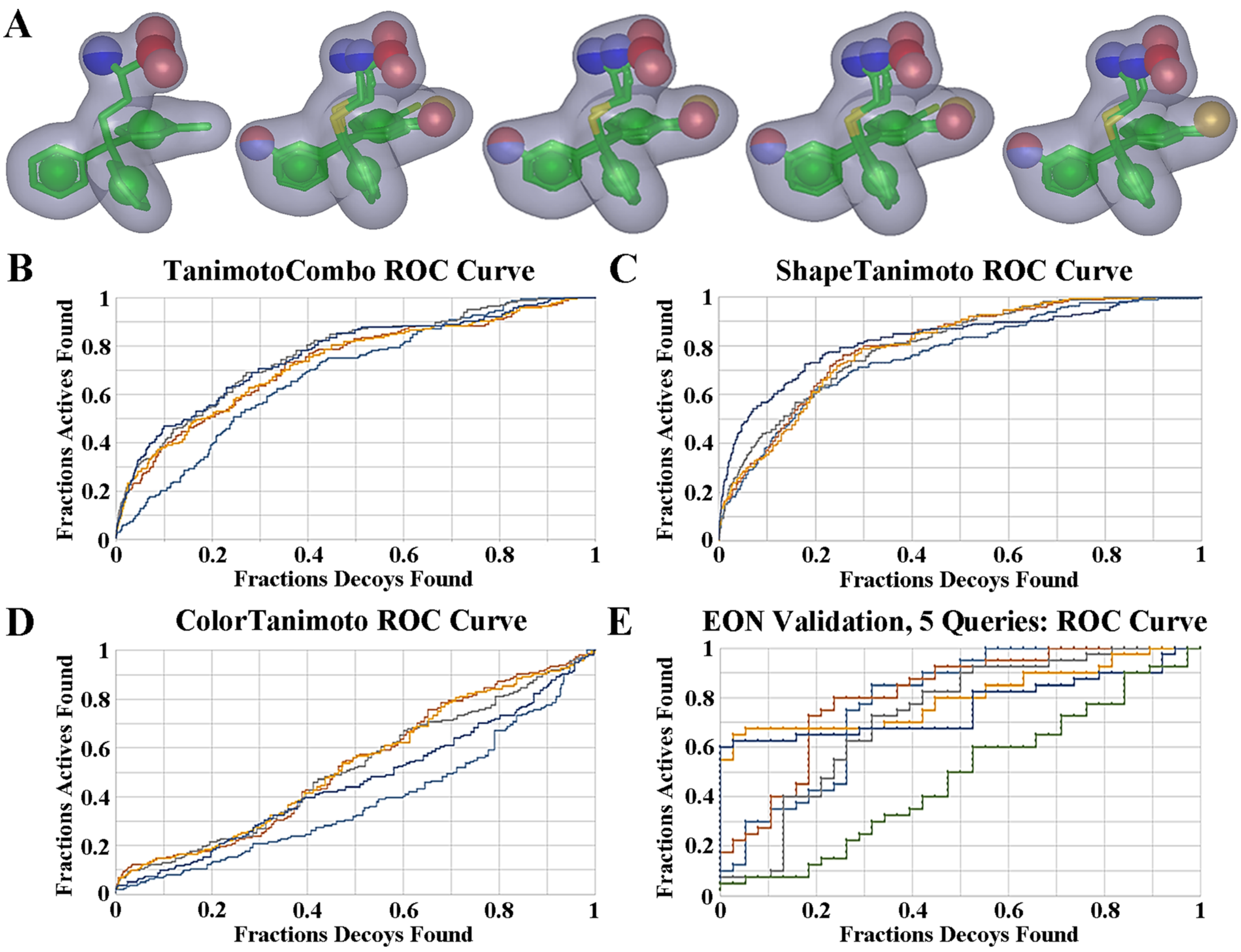

F

Query5 ShapeTanimoto (Average [95% Confidence])	
AUC	0.827 [0.788, 0.863]
0.5% Enrichment	28.765 [16.216, 43.709]
1.0% Enrichment	22.924 [16.168, 30.387]
2.0% Enrichment	15.885 [11.988, 20.000]

Supplementary Figure 2: ROCS and EON query validation ROC curves. (**A**) Five aligned queries generated by vROCS. From left to right: query 1 to query 5. (**B**) ROCS validation: ROC curve of five aligned queries. Scoring function: Tanimoto Combo. (**C**) ROCS validation: ROC curve of five aligned queries. Scoring function: Shape Tanimoto. (**D**) ROCS validation: ROC curve of five aligned queries. Scoring function: Color Tanimoto. In B-D, query 1 (light blue line), query 2 (red line), query 3 (grey line), query 4 (yellow line), query 5 (deep blue line). (**E**) EON validation: ROC curve of five single molecule queries. Scoring function: ET_Combo. 2XAE_2XA (ligand 2XA from crystal structure PDB ID: 2XAE, same pattern below, light blue line), 3KEN_ZZD (red line), 4A50_DQ6 (grey line), 4A51_DQ8 (yellow line), 4BBG_V02 (deep blue line), NO_EON (Shape Tanimoto score of hit list compounds, not rescored by EON, green line) (**F**) AUC and enrichment factors of query 5 using the scoring metric Shape Tanimoto.

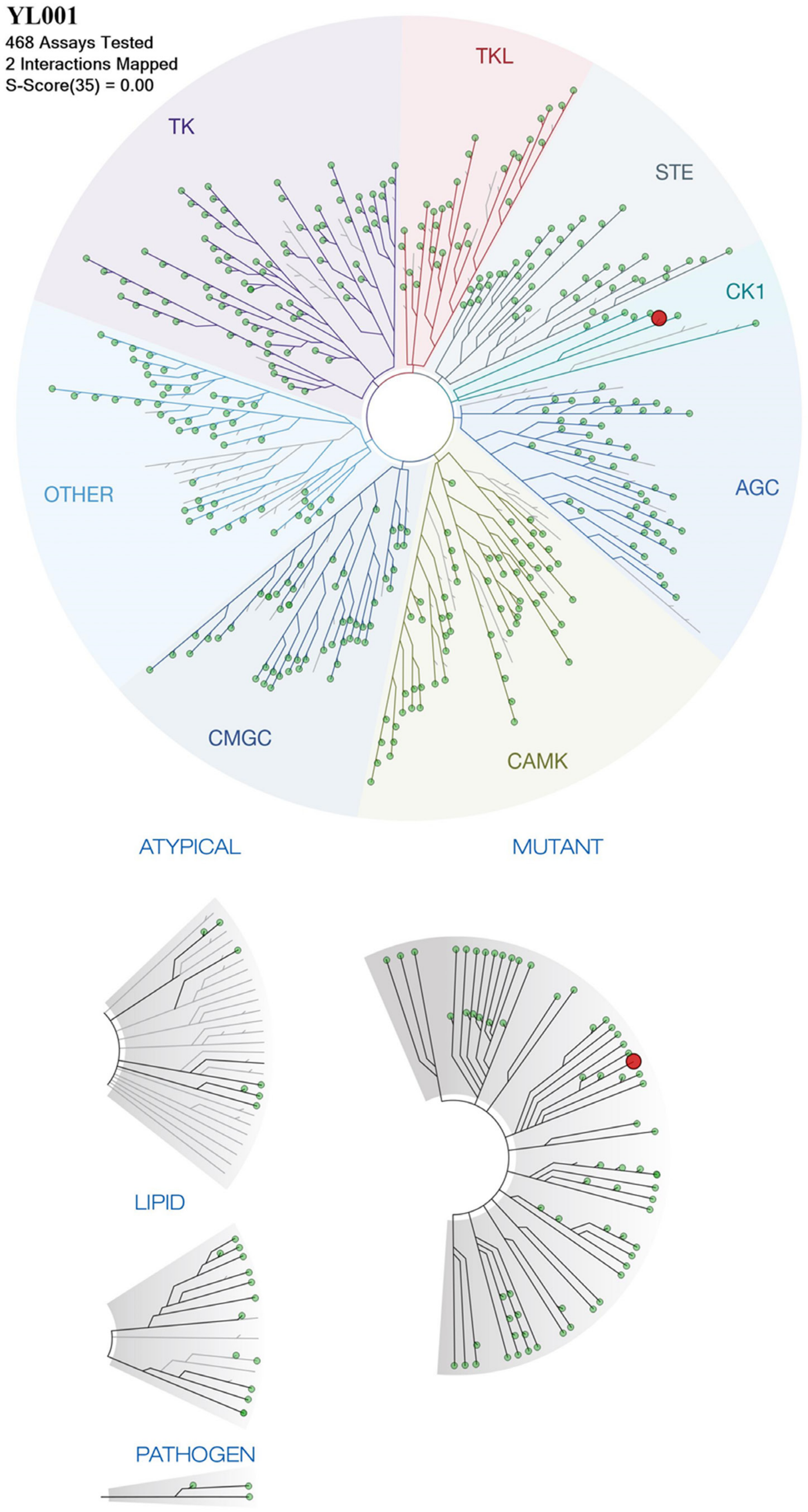

Supplementary Figure 3: Kinase Profiling data visualized as interaction tree maps. Kinase Profiling was used to evaluate activity of YL001 against a panel of 468 distinct human protein kinases, including typical, atypical, mutant ones. When YL001 inhibition to a kinase is over 35%, the kinase will be marked with red circle, where larger circle indicates higher-affinity binding.

Supplementary Table 1: SPR screening and antiproliferation screening of 23 manual selected compounds

Pick No.	Assay No.	Specs Compound ID	Binding Level (RU)	Inhibition (50 μM)
1	3	AG-401/37257005	18.8	95.06%
2	18	AG-205/13765047	3.4	–18.48%
3	12	AN-919/14547075	69.6	27.56%
4	16	AG-690/36921098	61.1	21.81%
5	11	AN-919/14547113	1.9	21.56%
6	15	AJ-292/15089069	100.1	58.97%
7	13	AN-919/14547078	56.6	30.07%
8	9	AN-465/13570058	7.4	98.24%
9	6	AG-227/37394007	8.7	97.78%
10	20	AK-778/11811129	59.8	9.38%
11	22	AH-487/42271989	31.9	41.63%
12	8	AN-919/14545008	57.7	26.79%
13	10	AG-205/14740101	27.7	0.87%
14	14	AF-399/14739190	36.9	22.02%
15	21	AF-399/41981012	3.8	14.85%
16	23	AP-906/42853823	60.7	19.16%
17	17	AE-842/33003047	14.6	–30.66%
18	2	AG-205/33162003	32.8	60.89%
19	7	AG-205/13460017	44.5	30.78%
20	5	AM-807/14957480	16.3	30.76%
21	4	AK-777/10805059	20.7	31.51%
22	19	AO-081/41364431	56.4	29.79%
23	1	AQ-714/41675002	2.3	32.65%
-	-	STLC	101.4	-

Proliferation inhibition was calculated with Alamar Blue. Binding level was evaluated with SPR. STLC was used as a control. Pick No. refers to the compound's EON ranking; the higher the ET_Combo, the lower the Pick No.

Supplementary Table 2: EC_{50}s (μM) of the YL001 in a breast cancer cell and a normal cell line

**Cell lines**	**Type**	**Source**	**EC_{50} of YL001 (μM)**
MCF-7	Epithelial	Breast cancer	14.90 ± 2.57
MCF10A	Epithelial	Breast normal cell	>100
Therapeutic window			>6.7

The therapeutic window is a ratio of the minimum effective concentration (EC_{50} on MCF-7) to the minimum toxic concentration (EC_{50} on MCF10A).
